# Self-regulated learning in physical therapy education: a non-randomized experimental study comparing self-directed and instruction-based learning

**DOI:** 10.1186/s12909-019-1484-3

**Published:** 2019-02-08

**Authors:** Wim van Lankveld, Marjo Maas, Joost van Wijchen, Volcmar Visser, J. Bart Staal

**Affiliations:** 1 HAN University of Applied Sciences, Research group Musculoskeletal Rehabilitation Nijmegen, Nijmegen, The Netherlands; 2 Radboud university medical center, Radboud Institute for Health Sciences, IQ healthcare, Nijmegen, the Netherlands

**Keywords:** Physical therapy education, Self-directed learning, Experimental study

## Abstract

**Background:**

There is a concern that traditional instruction based methods of learning do not adequately prepare students for the challenges of physical therapy practice. Self-directed learning is considered to be the most appropriate educational approach to enhance life-long learning as it enhances self-efficacy. This study compares outcomes in two educational approaches: self-directed learning (SDL), and traditional instruction based learning (IBL).

**Methods:**

In this non-randomized experimental study two groups of second year physiotherapy students were compared using pre-post-test assessments. Study results (both knowledge and physiotherapy performance), and self-reported self-efficacy were used as outcome variables. Study results from the end of year 1 and the end of year two were retrieved form the student information system. Self-reported variables including general and physical therapy self-efficacy were assessed using an online questionnaire which was completed at the start and the end of year two. Changes in self-efficacy were analysed using a repeated measures multivariate ANOVA.

**Results:**

A total of 174 students were enrolled in the second year, of which 108 (62%) agreed to participate in the online questionnaire. The online questionnaire at baseline (September 2015) was completed by 27 students in the SDL condition compared to 81 students in the IBL condition. There were no statistical differences at baseline between both educational approaches on any of the variables in the study. At the end of year two, there was no difference between both conditions in indicators of study results: knowledge and performance. Perceived self-efficacy in functioning as a physical therapist increased between both assessments. However, this increase was observed in both condition, and the difference between both conditions was not statistically significant.

**Conclusions:**

Self-directed learning and traditional instruction based learning result in equal study outcome and self-efficacy at the end of year two. More research is needed to determine the long term outcome that is most relevant for lifelong learning, and which students will benefit most from this approach. Nonetheless, self-directed learning might be an important alternative for instruction-based l education.

## Background

Physical therapy (PT) practice requires self-determined, professional clinical decision making in the face of an ever-increasing body of knowledge [1]. Rapid advances in medical science, technology, and changes in health care delivery pose a challenge to health care professionals [2]. To enable lifelong learning health professionals need to manage one’s learning by actively taking control of learning activities or self-regulated learning [3]. In PT education, instruction-based (IB) learning is the traditional didactical format, using conventional instruction in a classroom setting, in which a professional educator transfers knowledge or skills to the student according to an established time framed curriculum. There is a concern that these IB methods of learning do not adequately prepare students for the challenges of PT practice [4], and it has long been acknowledged that other modes of learning are needed in PT education [5]. Based on social constructivist, and social cognitive learning theories, educational approaches have been introduced, which emphasize the student’s active participation in learning, and develop knowledge and skills in the context I which it is applied [6]. In PT education contextual learning has been fully embraced, as it is emphasized in the frequent use of patient simulations and role playing [7–9]. Less attention has been given to the study of PT students active learning that is central to social constructivist theories of learning in higher education [2, 10, 11]. Social constructivism - as an educational philosophy - not only acknowledges the uniqueness and complexity of the learner, but actually encourages, utilizes and rewards this uniqueness as an integral part of the learning process [12]. Such an individualized approach, based on the uniqueness of the learner, emphasizing the individuals’ responsibility for the learning process is at the core of Self-Directed Learning [13–15]. Self-Directed Learning (SDL) is defined as ‘a process in which individuals take the initiative, with or without the help of others, in diagnosing their learning needs, formulating goals, identifying human and material resources for learning, choosing and implementing appropriate learning strategies, and evaluating learning outcomes’ [15]. A meta-analysis into studies evaluating SDL in medical education concluded that SDL in health professions education is associated with moderate improvement in the knowledge domain compared with traditional didactic teaching and may be as effective in the skills and attitudes domains [16]. SDL is considered by many to be the most appropriate approach for life-long learning [17]. SDL is not a homogeneous method and SDL can be operationalized in many ways [17]. Sometimes SDL is operationalized without teacher involvement, while other operationalisations include structured coaching by teachers.

One reason why SDL is considered effective for life-long learning is that it enhances the student’s self-efficacy beliefs [18]. According to Social Cognitive Learning Theory, behavior is motivated and regulated through external social systems and internal self-influencing factors [19, 20]. Self-efficacy (SE) is an internal self-influencing factor referring to an individual’s judgment of their capabilities to organize and execute courses of action required to achieve desired types of performances [19]. Self-efficacy is important both as a process and an outcome in learning. Self-efficacy for learning - referring to the students’ beliefs in the capability to regulate their own learning - is related to learning outcomes and academic achievement [21–23] . However, this relation is reciprocal as performance also reflects on the students’ learning self-efficacy [24]. Self-efficacy is thus highly relevant for student self-regulation, or the degree to which students are responsible participants in their own learning process [3]. Self-efficacy might also refer to the students’ confidence in his / her abilities to meet the challenges of their future profession. Therefore, self-efficacy in physical therapy practice, or task specific confidence, is considered critical to professional development of the novice PT [25–27], and is considered an independent predictor for student performance in clinical settings [27]. In line with Bandura’s theory [28] self-efficacy is domain specific. A recent study on Physical Therapy Self Efficacy (PTSE), showed that self-efficacy in the physical therapy domain was largely independent from study/work related self-efficacy [29]. Furthermore, within PT self-efficacy there is further domain specificity, with moderate correlations between PT self-efficacy beliefs in the musculoskeletal, neurological, and cardiovascular clinical conditions. PT students’ self-efficacy in each domain increases during their education as a PT and is related to the content of the education program [29].

Although self-efficacy has been at the center of social cognitive learning theory, there is only limited research that compares the effect of different educational approaches on self-efficacy in higher education [30, 31]. Studies comparing different educational approaches in Physical therapy education and their effect on Physical Therapy self-efficacy are, to our knowledge, not been conducted. Research is needed, as it has been argued that not all teaching techniques based on constructivism are efficient or effective for all learners in all situations [32]. Experiments conducted at a micro-analytic level are required when the aim is to detect causality, and further experimental research in specific settings is recommended [24].

Therefore we conducted a quasi-experimental study to compare the effects of two different educational approaches: a self-directed learning approach (SDL) and a traditional instruction-based approach (IB). In this study the students in the SDL approach are monitored by their teachers to help the student make the right choices.

Both conditions were compared on study performance, physical therapy self-efficacy, study related self-efficacy, and eagerness to study. It is assumed that it is more rewarding for the student if the student is responsible for the design of his own learning process, resulting in higher self-efficacy related to learning in the SDL condition approach.

## Methods

In this non-randomized experimental study two groups of physiotherapy students were compared using pre-posttest assesments. Both groups were taking part in the education programme at the HAN University of Applied Sciences, Nijmegen the Netherlands.

### Participants

Students enrolled in the second year of the bachelor Physiotherapy of the HAN University of Applied Sciences in Nijmegen, The Netherlands were asked to participate in this study. Students were invited by social media of the HAN University of Applied Science (email and face book), and only those students responding by email to the invitation were asked to complete the questionnaire after giving their informed consent. These students were invited to participate in a longitudinal study into changes in self-efficacy over one year. Participants were asked to complete an online questionnaire two times: at the beginning and the end of the study year 2. Student received information about this condition and the implications for their study. After giving their informed consent by checking the agree box in the web-based questionnaire, PT students were enrolled in the study.

### Allocation of students to condition

The experiment is limited to the second year of the physical therapy study. In the first year all students completed a structured IBL introduction curriculum. This curriculum also targets learning skills. To ensure that the student develops sufficient knowledge in year 1, he is periodically tested and receives feedback. As the completion of year 1 is a prerequisite to start in year 2, all students will be familiar with these learning skills and have similar levels of knowledge. The third year of the study includes a practice internship and is therefore not suitable for an experiment. Prior to the start of the second year, it was decided that the number of enrolled students would allow the formation of 7 classes. Two out of these 7 classes were selected to participate in the SDL condition. These classes were chosen by the two teachers who had developed SDL at HAN University of Applied Sciences. The same teachers (authors VV and JvW) would run the SDL condition together with two other teachers. At the start of study the second study year students of these two classes destined to take part in Self Directed Learning were extensively briefed about the new method of learning. After this briefing, students were allowed to switch to the traditional instruction-based program prior to the start of the curriculum.

### Conditions

For both conditions the program outcomes were identical and determined in advance. Furthermore, all students were submitted to the same exam and evaluation regime. Students were provided with an assessment manual containing the learning content of the educational program, as well as the assessment formats. In this way, students new in advance how their levels of knowledge and competence will be assessed at the end of the year. Table 1 shows how the didactics differ between condition 1 and 2.

**Table 1:** Differences between Self-Directed and Instruction Based Learning

Program	Self-directed learning (1)	Instruction-based learning (2)
Program outcomes	Pre-defined	Pre-defined
Learning goals	Self-directed for each program activity	Pre-defined for each scheduled program activity
Learning content	Self-directed for each program activity	Preset for each scheduled program activity
Learning activities	Self-directed for each program activity, no teacher manual.	Instruction based for each scheduled program activity, supported by a teacher manual.
Role of teacher	Coach in choosing relevant personal learning goals and learning activities. Providing performance feedback.	Coach in guiding learning activities towards pre-defined learning goals. Providing performance feedback.
Monitoring learning progress	Student in the lead	Teacher in the lead
Assessment of learning outcomes.	Pre-defined	Pre-defined
Assessment criteria and procedure	Pre-defined	Pre-defined

#### The self-directed learning condition (SDL)

At our institution, SDL is organized in a community of practice involving teachers and students [33]. In this community of practice, responsibility for the learning outcomes is shared among all actors. The desired minimum competence outcomes of the learning program and the related assessments are pre-defined. With these outcomes in mind, the students are involved in identifying their learning needs and develop learning activities based on their individual needs and self-drive [17]. As a result the learning content and learning activities are tailored to the individual student’s needs and take highly individualized and distinct forms.

The teacher guides the student through this complex process. The role of the teacher within this community is firstly to challenge students to set their own goals, to design their own learning tasks and to plan their own learning activities. Second, the teacher is a coach, providing students with demand-oriented performance feedback. Third, the teacher is a monitor, evaluating the individuals’ learning progress.

#### Instruction based learning (IBL)

In this condition the program is highly structured week by week in a classroom setting. It contains pre-defined learning goals, learning tasks, learning content, and learning outcomes week by week. Every student follows the same learning trajectory. The role of the teacher is first an instructor, clarifying learning goals and providing learning tasks. Second, the teacher is a coach, providing task-oriented performance feedback.

### Measurements

Study results of all students were retrieved from our institutions student information system. Student self-reported variables including informed consent were gathered using a web based programme. Once logged in, the students were required by the system to answer every question thus preventing missing items. Students were asked to complete the online questionnaire twice: the first time in week 2 of the second year, the second time in the last week of the study year.

*Baseline characteristics:* gender, age, prior level of education, voluntary work (yes/no), paid jobs (yes/no), and weekly hours spend on sporting activities.

### Outcome measures

*Study results* retrieved from our institutions student information system included assessments of knowledge and clinical performance in a simulated setting. Knowledge is an essential basis for physiotherapy practice. Knowledge is explicitly tested by four knowledge tests every year. A knowledge test is an online standardized test consisting of 80 digitally presented closed questions related to the content domains of anatomy, physiology, pathophysiology, motor learning and movement, behavior and communication. All students are required to complete the same test at the same moment under controlled conditions. Questions can measure replication of knowledge, understanding of knowledge, or application of knowledge. A variety of stimulus- and response formats is used (yes/no questions, multiple choice questions, best choice questions, key-feature t questions). Written questions can be illustrated by pictures, photographs or video-recording. Correct item scores were described, sum scores and mean scores were calculated, and final scores were expressed on a scale from 0 to 10, with 10 the maximum score of correct answers. Clinical performance is assessed in a simulated setting with standardised patients. Standardised patients are instructed to perform written clinical cases relevant to the content of the course. Performance assessors use global performance indicators and are trained in their assessor role in calibration sessions using video-recordings of performances. In this study, performance assessors worked in couples to enhance objectivity and were blinded for SDL or IBL students. Students were asked to demonstrate the physiotherapy intake, a relevant part of the clinical examination to diagnose the problem, and a relevant part of an intervention to enhance recovery. .

*Self-efficacy related to work/study* is assessed using the self-reported Psychological Capability scales (PsyCap). The PsyCap measures self-efficacy related to work/study in four distinct dimensions: self-effectivity, hope, optimism, and resilience. [34, 35]. PsyCap consists of 22 items to be scored on a six point Likert scale (1 = strongly disagree; 6 = strongly agree). Self-effectivity in the PsyCap is defined as an individual’s confidence in their ability to mobilize their motivation, cognitive resources and courses of action to achieve high levels of work related performance [36]. Higher scores reflect higher levels of psychological capability. For each subscale, the average items score was computed.

*Self-efficacy related to working as a physical therapist* was assessed using the Physical Therapy Self Efficacy (PTSE). The PTSE measures self-efficacy beliefs in three clinical areas with 39 five point Likert items [29]. The participants were asked to indicate their confidence to perform 13 PT tasks for the musculoskeletal, neurological, and vascular clinical conditions (1 = very little confidence; 5 = a lot of confidence). The instrument takes on average 10 min to complete. For each subscale, the average items score was computed.

*Eagerness to study* was assessed using three items to determine the student’s motivational attitude towards the study. The three items are: “I like to study more than absolutely necessary”, “In enjoy learning”, and “I actively seek new challenges in studying”. Items were constructed by the researchers, based on the teacher’s evaluation of important topics in student motivation of learning. Items were scored using a 6 item Likert scale (1 = completely disagree; 6 completely agree. Average item score for the three items was calculated to reflect Eagerness to study (cronbach’s alpha = 0.67).

### Data analysis

Descriptive statistics of student samples for ordinal and nominal data are given including proportions. Because self-reported online questionnaire were conducted on a voluntary basis, not all students in study year 2 participated in the study. To determine whether agreement to participate in the study constituted a selection bias the groups of students participating in the study were compared with the group of students declining to participate. To this end, study results in the year prior to the experimental year were retrieved from the student information system. Average scores of knowledge and performance test of the last semester of year 1 were calculated for both groups to determine whether the groups differed in study performance at the start of year 2. Differences were analysed using Chi-square for discreet, and independent samples T tests for continuous variables. In the same way differences in baseline characteristics between students in the study allocated to the SDL en IBL conditions were compared. In addition to study performance, self-reported baseline outcome measures were compared. For the 3 item Eagerness to study scale associations with measures of self-efficacy were analysed using pearson correlations (r). The strength of correlations is defined as negligible (0.00 to 0.30), low (0.30 to 0.50), moderate (0.50 to 0.70), high (0.70 to 0.90), very high (.90 to 1.00). Next, differences between both conditions at the end of year two were compared. First, differences in study performance between both conditions were analysed by comparing study performance at the end of year 2. Next, differences between the two teaching methods during the year of study in changes in self-efficacy and eagerness to study were tested using a GLM repeated measurement design. A repeated measures multivariate ANOVA was conducted with the three PTSE subscales as independent variables and baseline and follow up assessment defining time variate. Multivariate F values for the within subjects effects time and time * groups effects are reported with degrees of freedom in brackets (hypothesis degrees of freedom, error degrees of freedom), as well as significance level. Difference between both conditions in study related self-efficacy and Eagerness to study was analysed in a similar way with PyCap (4 subscales) and Eagerness to study as dependent variables. For these analysis, only students that completed both self-report questionnaires were included.

The Statistical Package for the Social Sciences (SPSS) version 21 was used for statistical analysis, and a value of *p* < .05 was considered statistical significant.

## Results

**Fig. 1:**
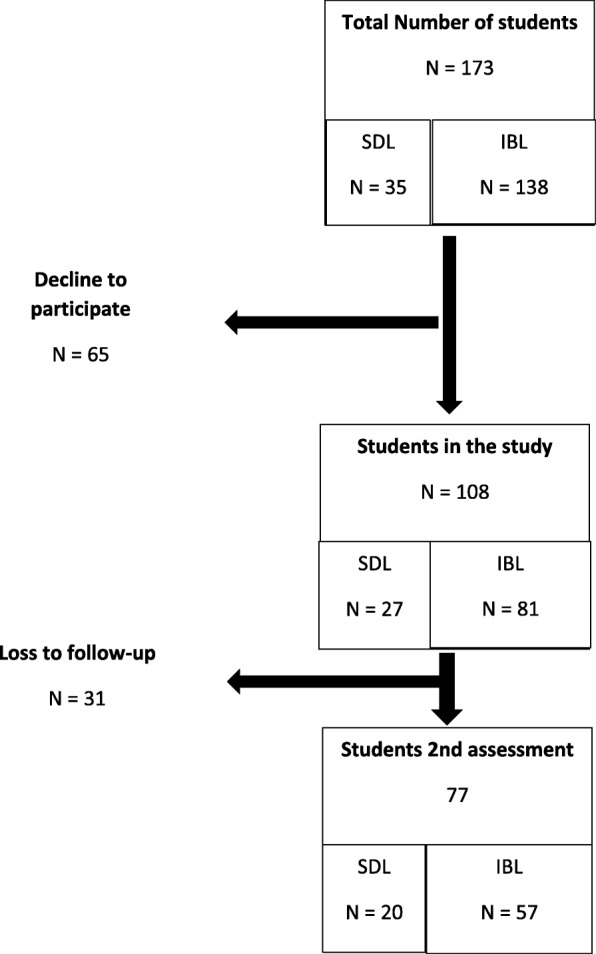
Flow chart of students and conditions

In Fig. 1 (flowchart), the number of students at every step of the study is depicted. A total of 173 students were enrolled in the second year, and 35 were allocated in the SDL condition and the remaining 138 in the IBL condition. Before the start of the curriculum, 3 male students in the determined SDL condition switched to IBL after the presentation to the selected classes. A total of 108 students (61%) agreed to participate in the online questionnaire. The online questionnaire at baseline (September 2015) was completed by 27 students in the SDL condition compared to 81 students in the IBL condition. The proportion of students completing the questionnaire was higher in the SDL condition compared to the IBL condition (77% versus 56%, Chi-square = 4.8, *p* < 05). The proportion of male / female in the participants in the study was 42/73. The average age was 19.8 years (range 17–29, SD = 2.7). Of these students, 33% was living in a student house (dormitory), 4% together with a partner, and 61% was living with their parents. Voluntary work was done by few students (15%), compared to a paid job in 75% of the students. With the exception of three students, all were engaged in sporting activities.

The group of students participating in the study showed higher average study results in the previous semester compared to students not participating in the study. Average Knowledge assessment was 6.7 (SD 0.7) in the participants compared to 6.4 (0.5) in the non-participants (T = 2.2, df = 170, *p* < .05). Average performance assessment was 7.2 (SD 1.0) in the participants versus 6.7 (SD 0.7) in the non-participant group (T = 2.7, df = 170, *p* < .05).

Next, the differences at baseline between the SDL and IBL conditions in study results in the previous semester and self-reported outcome measures online were computed. In Table 2 group baseline characteristics of both conditions are given.

**Table 2:** Student characteristics at baseline

	Condition 1 (*N* = 27)	Condition 2 (*N* = 79)
Average Age (SD)	19.5 (1.9)	20.1 (2.9)
Gender (% female)	67%	62%
Living situation (% living with their parents)	68%	58%
Part time jobs	77%	73%
Active in sports	97%	98%
Study results knowledge year 1	6.8 (0.7)	6.7 (0.7)
Study results performance year 1	7.1 (1.1)	7.3 (1.0)
PTSE Cardiovascular (range 1–5)	3.0 (0.73)	3.2 (0.67)
PTSE musculoscelethal (range 1–5)	3.6 (0.68)	3.7 (0.52)
PTSE Neurological (range 1–5)	2.7 (0.76)	2.8 (0,88)
PsyCap Self-efficacy (range 1–6)	4.3 (0.75)	4.5 (0.57)
PsyCap Hope (range 1–6)	4.6 (0.81)	4.7 (0.58)
PsyCap Optimism (range 1–6)	3.8 (0.45)	3.9 (0.51)
PsyCap Resilience (range 1–6)	4.0 (0.54)	4.1 (0.45)
Eagerness to study (range 1–6)	4.6 (0.70)	4.4 (0.64)

No significant differences between groups were found in any of the demographic variables. Including average age and gender. Average study results in both knowledge and performance assessment in the last semester of year 1 were similar in both groups. No differences were found in average scores in the self-efficacy scales (PTSE, and PsyCap sub scale) or eagerness to study. Comparing the different PTSE subscales showed that musculoskeletal caseload self-efficacy was higher compared to self-efficacy related to other caseloads (average score respectively 3.7, 3.1, 2.7; Anova of Kendall’s W *p* < .001). At baseline, eagerness to study showed low correlation with the PsyCap subscales self-efficacy (r = 0.32, *p* < .000), hope (r = 0.37, *p* < .000), and resilience (r = 0.21, *p* < .05). The PsyCap Scales have no significant correlations with the PTSE.

### Study results at the end of year 2 compared

There was no statistical difference between average scores in both conditions on either knowledge or performance assessment at the end of year two. Average scores on the knowledge test was 6.3 (SD 0.67) in the SDL compared to 6.2 (SD 0.71) in the IBL condition (T = 0.4, ns). Average performance was 6.75 (SD = 0.9) and 6.7 (SD = 0.9) in the SDL and IBL conditions respectively (T = 0.4, ns).

### Changes in physiotherapy self-efficacy during year 2

Self-reported assessments at time 2 was completed by 20 students in condition 1, and 57 students in condition 2. Students not completing the second measurement did not differ from student completing both assessment on any of the baseline characteristics. Table 3 shows average item scores for the PTSE subscales for both groups on both assessments.

**Table 3:** average score in PTSE subscale scores at Time 1 (start of year 2) and 2 (end of year 2)

		Time 1 Mean (sd)	Time 2 Mean (sd)
neurological	Condition 1	2.75 (0.75)	3.41 (0.55)
	Condition 2	2.82 (0.77)	3.57 (0.48)
	Average	2.79 (0.76)	3.53 (0.51)
musculoscelethal	Condition 1	3.62 (0.58)	3.77 (0.38)
	Condition 2	3.79 (0.43)	3.98 (0.34)
	Average	375 (0.48)	3.92 (0.37)
cardiology	Condition 1	2.86 (0.69)	3.63 (0.39)
	Condition 2	3.06 (0.65)	3.64 (0.44)
	Average	3.00 (0.66)	3.63 (3.64)

PTSE = Physical Therapy Self Efficacy, SD = Standard deviation

In the repeated measures ANOVA there was a time effect within subjects effect on physical therapy self-efficacy (Multivariate F (3,72) = 28.8, *p* < .0001). The interaction effect of time and groups was not significant (Multivariate F (3,72) = 1.09, p = n.s.). That is, physical therapy self-efficacy changed over time in both conditions and that change was similar in both conditions. Univariate testing showed that scores on all three PTSE sub-scales increase over time (Mean difference Neuro = 0.72, *P* < .001, 95% CI mean difference = 0.53,0.89, F = 70, *p* < .01 Mean difference musculoskeletal = 0.17, *p* < .05, 95% CI mean difference = 0.04, 0.3, F = 7.0, p < .05; Mean difference cardiovascular 0.67, 95% CI mean difference = 0.51, 0.83, F = 58.8, p < .01). The increase in self-efficacy was most pronounced on the scale Neurological Casualty (from 2.8 to 3.5, an increase of 1 SD compared to the pre-measurement).

### Changes in PsyCap and eagerness to study

In a similar way the change in PsyCap and Eagerness to study were analysed using a multivariate test-retest ANOVA. Results are depicted in Table 4.

**Table 4:** PsyCap scales and Eagerness to study at baseline and follow up for both conditions

		Time 1	Time 2
		Mean (sd)	Mean (sd)
Self-efficacy	Condition 1	4.3 (0.6)	4.4 (0.6)
	Condition 2	4.6 (0.6)	4.9 (0.51)
	Total	4.5 (0.57)	4.6 (0.56)
Optimism	Condition 1	3.8 (0.56)	3.8 (0.48)
	Condition 2	3.8 (0.50)	3.8 (0.42)
	Total	3.8 (0.50)	3.8 (0.44)
Resilience	Condition 1	4.0 (0.65)	4.1 (0.40)
	Condition 2	4.1 (0.48)	4.2 (0.43)
	Total	4.0 (0.53)	4.2 (0.42)
Hope	Condition 1	4.3 (0.83)	4.7 (0.47)
	Condition 2	4.6 (0.59)	4.7 (0.38)
	Total	4.6 (0.67)	4.7 (0.40)
Eagerness	Condition 1	4.6 (0.65)	4.6 (0.70)
	Condition 2	4.5 (0.60)	4.4 (0.64)
	Total	4.5 (0.61)	4.5 (0.66)

In the repeated measures ANOVA the main within subjects effect of time was not-significant (Multivariate F value = 1.15; df = 5, *p* < .34). The group effect was not significant in the multivariate analysis (Multivariate F = 0.8, df = 5, *p* = .07). For the PscyCap and Eagerness to study scales, there was no change over time, and no difference between the two groups.

## Discussion

This is the first study comparing the effect of PT training in a guided self-directed learning approach, emphasizing the students responsibility for their own learning process, to a traditional, structured classroom learning situation. Students in both conditions showed equal study performance at the end of the study. SDL did not result in higher self-efficacy, either PT specific or self-efficacy related to learning. The belief to be able to function as a physical therapist (physiotherapy self-efficacy), did increase between both assessments, but both conditions were equally effective in enhancing physical therapy self-efficacy. Participating in different learning conditions did not have an impact on self-efficacy beliefs related to learning or Eagerness to study. Both conditions performed equally well in all outcome measures. These findings are in line with one systematic review into physiotherapy education, concluding that no model of clinical education or physiotherapy students is superior to another [37]. Another important finding of this study is that, perceived self-efficacy related to learning did not change over time, whereas self-efficacy beliefs about functioning as physical therapist increased over time. Self-efficacy beliefs about learning in students is related to study success [21–23]. However, the direction of causality is not always clear. The relation between self-efficacy and study success is reciprocal, and the experience of earlier study success might enhance self-efficacy beliefs [24]. Nonetheless, self-efficacy beliefs about learning are highly relevant for student self-regulation, or the degree to which students are responsible participants in their own learning process [3]. However, the assumption that the SDL condition approach it is more rewarding for the student if the student is responsible for the design of his own learning process was not reflected in an increased self-efficacy related to learning in this study. The findings with regards to the increase of self-efficacy beliefs related to functioning as a physical therapist underline Bandura’s suggestion that self-efficacy is domain specific [19]. Measuring perceived domain specific self-efficacy is likely to be more informative for both the students and the teacher. Because increase in PT self-efficacy was similar in both conditions it can be concluded that PT self-efficacy increases regardless of condition.

This study has some mayor drawbacks. One important drawback is that it is not clear how exactly the two classes were chosen to be included in the SDL condition. Two of the authors (VV and JvW) volunteered to develop and implement SDL, and in their role as teachers, they selected the groups (classes) to be allocated to the SDL condition. This selection of groups by the teachers might pose a selection bias, increasing the change that the selected groups perform better compared to the non-chosen groups. Furthermore, students allocated to SDL were allowed to switch to the IBL condition after being instructed on SDL. Comparison of both conditions on baseline and follow-up assessment did not show any significant differences, refuting the impact of selection bias. However, it cannot be ruled out that there are differences in both conditions not operationalized in this study. To eliminate confounding of indication, a randomized trial in which students are randomly allocated to the teaching intervention could be considered. However, such a forced allocation is at odds with self-determination principles and difficult to organize and implement. Another drawback from this study is that not all student participated in the study. Those student volunteering to participate had, on average, higher study results, compared to the students not participating in the study. Therefore, it is not clear whether these findings can be generalized to all students. Furthermore, not all students entering the study completed the second assessment, posing a possible second selection bias (loss to follow up). However, comparing the students completing both assessments with those students not completing both assessments did not result in any difference, on any of the baseline variables. Finally, we did not measure the effect of the two conditions on functioning in the clinical phase of the study, or later on in their professional career. As a consequence, it is not possible to draw conclusion about the effect of the intervention on the students lifelong learning ability in new situations.

Nonetheless, this quasi experimental study shows that self-directed learning in PT education is possible, and that it does not lead to lower study results. As such, SDL is a valuable addition in the differentiation of learning methods. Although self-directed learning has been propagated by some in higher education, this methods is not without its criticism [38–40]. In particular, self-directed learning asks for high levels of reflective skills and meta cognitive learning [41]. Furthermore, many students develop faulty mental models of how they learn, resulting in inappropriate judgement of learning [40]. Therefore, it has been argued that not all teaching techniques based on constructivism are efficient or effective for all learners in all situations [38]. This was evident in our study where 3 students switched between conditions, indicating that some students preferred IBL to SDL.

Therefore, future research into the differentiation of learning should focus on the matching of PT students to different educational approaches. Such an approach would need to assess and address the individuals students beliefs and illusions about SRL [40], and determine the student’s willingness or readiness to engage in SDL [42]. In medical and nursing education, such studies have been reported using self-reported questionnaires [42, 43]. However, such studies have not been conducted in PT education. Furthermore, the constructivist approach to learning also has implications for the evaluation of learning and the evaluation of SDL in particular. Acquisition of knowledge and/or skills is not sufficient to be prepared for an unknown future. This means that, apart from knowledge and skills, other values like self-efficacy should be considered in evaluating the outcome of an education. For those scholars unfamiliar with the topic, guidance to scholars on how to improve, evaluate, and study self-effectivity have been made available [23]. Finally, it is worthwhile to investigate what the effect of the different educational approaches is on performance during practice internship. Overall, there is a need for more research in constructivism based education because these approaches as yet lack evidence based support [44].

## Conclusions

Self-directed learning is a viable option in PT education. Self-directed learning and instruction based learning have similar study results in PT students, with similar impact on PT self-efficacy. However, as yet it is unclear which patients are likely to benefit most of self-directed learning.

## Abbreviations

GLM: General Linear Model
IBL: Instruction Based Learning.
PsyCap: Psychological Capability
PT: Physical Therapy
PTSE: Physical Therapy Self Efficacy
SDL: Directed learning.
SE: Self Efficacy
